# Taking another look at intelligence and personality using an eye-tracking approach

**DOI:** 10.1038/s41539-024-00252-8

**Published:** 2024-07-01

**Authors:** Lisa Bardach, Aki Schumacher, Ulrich Trautwein, Enkelejda Kasneci, Maike Tibus, Franz Wortha, Peter Gerjets, Tobias Appel

**Affiliations:** 1 Department of Psychology, University of Giessen, Giessen, Germany; 2 Hector Research Institute of Education Sciences and Psychology, University of Tübingen, Tübingen, Germany; 3 Technical University of Munich, Munich, Germany; 4 Loughborough University, Loughborough, UK; 5 Leibniz Institut für Wissensmedien, Tübingen, Germany

**Keywords:** Human behaviour, Human behaviour

## Abstract

Intelligence and personality are both key drivers of learning. This study extends prior research on intelligence and personality by adopting a behavioral-process-related eye-tracking approach. We tested 182 adults on fluid intelligence and the Big Five personality traits. Eye-tracking information (gaze patterns) was recorded while participants completed the intelligence test. Machine learning models showed that personality explained 3.18% of the variance in intelligence test scores, with Openness and, surprisingly, Agreeableness most meaningfully contributing to the prediction. Facet-level measures of personality explained a larger amount of variance (7.67%) in intelligence test scores than the trait-level measures, with the largest coefficients obtained for Ideas and Values (Openness) and Compliance and Trust (Agreeableness). Gaze patterns explained a substantial amount of variance in intelligence test performance (35.91%). Gaze patterns were unrelated to the Big Five personality traits, but some of the facets (especially Self-Consciousness from Neuroticism and Assertiveness from Extraversion) were related to gaze. Gaze patterns reflected the test-solving strategies described in the literature (constructive matching, response elimination) to some extent. A combined feature vector consisting of gaze-based predictions and personality traits explained 37.50% of the variance in intelligence test performance, with significant unique contributions from both personality and gaze patterns. A model that included personality facets and gaze explained 38.02% of the variance in intelligence test performance. Although behavioral data thus clearly outperformed “traditional” psychological measures (Big Five personality) in predicting intelligence test performance, our results also underscore the independent contributions of personality and gaze patterns in predicting intelligence test performance.

## Introduction

Intelligence and personality traits are both important predictors of learning^1,2^, and researchers have long been interested in how intelligence and personality are related^3^. However, studies on intelligence and personality have yet to consider the role of behaviors in intelligence test situations, even though various technologies (e.g., eye-tracking) offer opportunities to unobtrusively capture rich behavioral process data while individuals take intelligence tests^4–6^. Combined with advanced machine-learning-based analyses^7^, these technological applications make it possible to examine intriguing questions that go beyond linking personality scores and intelligence test scores. For example, what do people do while working on intelligence tests, that is, can we differentiate people who do well versus less well on the basis of their behaviors (e.g., gaze patterns) while they take an intelligence test? What are characteristics of such gaze patterns (i.e., do they reflect specific test-solving strategies)? How is personality associated with gaze patterns? And, finally, to what extent do personality traits and gaze patterns uniquely predict intelligence test scores?

With the present study, we set out to address all these questions by pairing behavioral data (eye-tracking) with “traditional” psychological measures (fluid intelligence, Big Five personality traits). Specifically, we aimed to examine relationships between the Big Five personality traits (Openness to Experience, Conscientiousness, Extraversion, Agreeableness, and Neuroticism) and fluid intelligence test scores, investigate whether gaze patterns predict intelligence test scores and personality traits, and identify specific intelligence-test-solving strategies on the basis of gaze patterns. We also looked at the Big Five facet scores which capture more specific aspects of each of the five broader personality traits. Lastly, we aimed to explore whether personality traits (and facets) add to the prediction of intelligence test scores while accounting for the variance explained by gaze patterns. As prior research on gaze patterns while individuals worked on intelligence tests has largely been based on untimed tests^4,6,8,9^, another novel contribution involves the use of a timed intelligence test in our study. The context of our study thus more closely resembles “typical” intelligence test situations in applied settings, which often rely on timed tests for practical reasons. The remainder of the introduction section is structured as follows. First, we provide an overview of research on personality-intelligence associations. Next, we discuss the promise of eye-tracking to provide a behavioral-process-related perspective on intelligence test performance, and cover relationships between personality traits and gaze patterns. We then present the research questions addressed in our study.

Intelligence is commonly defined as general mental capability^10^, with many scholars further distinguishing between the two categories of fluid intelligence (the capacity to solve novel and abstract problems), which we focused on in our study, and crystallized intelligence (general knowledge resulting from learning experiences^11,12^). Personality traits, on the other hand, capture relatively enduring patterns in how people think, feel, and behave^13^. Most research on intelligence–personality associations has adopted the Big Five model^14,15^, which organizes personality along the five dimensions of Openness to Experience (e.g., open-minded, curious, cultured), Conscientiousness (e.g., self-controlled, abiding by rules, organized), Extraversion (e.g., active, social, assertive), Agreeableness (e.g., cooperative, altruistic, tender-minded), and Neuroticism (e.g., anxious, worried).

Openness, as primarily a cognitive trait^16^, has received the most theoretical and empirical attention in research on intelligence–personality associations^17^. Investment trait theory suggests that so-called investment traits (e.g., Openness to Experience) determine the time and effort individuals invest in their intellect (e.g., by seeking out learning opportunities and intellectually stimulating environments). This investment presumably positively affects their intellectual development and underlies positive links between Openness and intelligence^18–20^. A recent meta-analysis^21^ revealed that Openness was the Big Five trait that was most strongly correlated with fluid intelligence (*r* = 0.170).

The second Big Five trait that has been highlighted in research on the interface between personality and intelligence is Neuroticism. Individuals scoring high on Neuroticism may, for example, exhibit higher levels of test anxiety, which may cause them to underperform on intelligence tests^22^. Neuroticism has been found to be significantly negatively correlated with fluid intelligence (*r* = −0.102)^21^. Relationships between the other Big Five traits (Extraversion, Agreeableness, Conscientiousness) and intelligence have generally been found to be weaker and less consistent^21,23,24^.

Overall, mounting evidence indicates that relationships between personality and other variables, including intelligence, are best understood on the facet or even the item level^21,25,26^. With respect to facets, the strongest relationships with fluid intelligence test scores have been obtained for Openness facets (especially Ideas, *r* = 0.253, and, to a lesser extent, Values, *r* = 0.153, and Fantasy, *r* = 0.136). Several Neuroticism facets have also been found to be significantly correlated with fluid intelligence (Anxiety, Depression, Vulnerability, and Angry Hostility, with *r*s ranging from −0.065 to −0.086). Some facets of Agreeableness (Modesty, Tendermindedness, Trust), Extraversion (Warmth, Gregariousness), and Conscientiousness (Competence) displayed significant yet negligible relationships with fluid intelligence as well^21^.

Complementing the large body of research linking intelligence with other determinants of individual differences (e.g., personality) or learning outcomes, another line of research has contributed to knowledge about intelligence test performance by taking a closer look at the test situation. Here, eye-tracking is promising, as it allows researchers to track eye behaviors (gaze patterns) while individuals work on intelligence tests.

Gaze information has successfully been used to predict intelligence test performance^27^. Hence, individuals who are more versus less successful in solving intelligence test items differ in their gaze patterns. In addition, to gain a better understanding of the specific gaze characteristics that drive intelligence test performance, scholars have, for example, used gaze patterns to trace test-solving strategies^4,6,28^. Research on fluid intelligence tests with multiple response options where participants have to choose the missing piece from several alternatives in a visual display (e.g., matrix reasoning tasks) has primarily focused on two types of strategies: constructive matching and response elimination^8,29,30^ (for extensions, see, refs. ^31,32^). Constructive matching involves the mental construction of the answer prior to searching for the match from among the response options. By contrast, when employing the less systematic strategy of response elimination, individuals frequently switch back and forth from the question to the response options to determine which options can be rejected^28,32^. Systematic individual differences in the tendency to use constructive matching and response elimination have been observed. Individuals with higher cognitive abilities tend to demonstrate more constructive matching, which has been found, to some extent, to explain their better performance^30,33^.

Furthermore, prior research using eye-tracking to explore individuals’ gaze patterns while working on intelligence tests has largely been based on unspeeded versions of these tests^4,6,8,9^. Hence, research has yet to test the extents to which (a) gaze patterns predict intelligence also on speeded tests and (b) gaze patterns indicative of certain strategies (constructive matching, response elimination) can also be identified on speeded tests. As research in applied settings (e.g., schools or other learning settings; tests administered as part of selection procedures) often relies on timed tests for practical reasons, the generalizability of prior findings to speeded test conditions is an important question.

Various ideas about how gaze patterns are related to intelligence test performance exist. Gaze patterns can index underlying cognition; hence, certain gaze patterns are behavioral manifestations of intelligence test performance. In research on how to train people to increase their fluid intelligence (and especially by critics of such studies), it has also been suggested that certain gaze patterns (reflecting specific test-solving strategies) can, to some extent, “cause” performance on intelligence tests, as individuals can be instructed to use certain strategies to increase their performance on the test^5,34^. According to this second view, gaze patterns pick up additional performance elements to some extent. The two perspectives (gaze patterns index underlying cognition; gaze patterns cause performance) are likely entangled. To illustrate, an individual may use a certain gaze pattern to solve item x successfully, which prompts them to adopt the same strategy for item x + 1. If two people with different intelligence levels then both use—or are instructed to use—this same strategy, the successful adoption of this strategy may be predictive of how well they do. At the same time, strategy use and actual ability are not independent, and particularly once intelligence test items become more difficult, more complex strategies (e.g., constructive matching) will only be accessible to those with higher levels of cognitive abilities: Mentally constructing the correct solution prior to looking at the response options and then just searching for this solution in the response options will not work if the person has no idea what the correct solution might look like (i.e., if they lack the cognitive abilities to mentally construct at least a somewhat suitable response option by themselves). Overall, zooming in on the role of gaze patterns during intelligence test completion allows researchers to gain a better understanding of the behaviors undergirding individual differences in fluid intelligence test performance^32^.

Gaze patterns may, to some extent, reveal individuals’ intelligence test performance, but do these patterns also provide information about personality? Even though research on personality has in general long called for a greater focus on actual behaviors^35^, no studies have, to the best of our knowledge, adopted eye-tracking to link personality traits to gaze patterns while individuals take an intelligence test. Nonetheless, even though Gonthier and Roulin^30^ did not include eye-tracking, they found that Typical Intellectual Engagement, a personality trait conceptually close to some aspects of Openness to Experience, predicted greater reliance on constructive matching as assessed via verbal reports on a matrix reasoning task. It appears reasonable to assume that personality traits that are more closely related to intelligence test performance should also be more strongly related to gaze patterns that predict intelligence test performance^30^. Openness to Experience is not only the Big Five personality trait that is most proximal to cognitive functioning; it has, for example, also been linked to adaptive learning approaches (e.g., deep learning strategies^36^). Therefore, scoring high on Openness to Experience could predict gaze patterns that are conducive to intelligence test performance. By contrast, not only should high levels of Neuroticism interfere with intelligence test performance^22^, but Neuroticism could also manifest in less beneficial gaze patterns.

Further, if personality traits and gaze patterns are considered simultaneously to predict intelligence test performance, it seems likely that personality can explain a unique amount of variance not captured by gaze patterns (and vice versa). Whereas gaze patterns are specific to the test situation and represent what individuals do while taking the test, personality is much broader, with some personality traits shaping (and being shaped by) individuals’ long-term cognitive development in real-world contexts (for Openness to Experience, see, e.g., intellectual investment trait theory’s assumptions). Therefore, Big Five personality traits (e.g., Openness to Experience, Neuroticism) and their respective facets should carry some distinct information for predicting intelligence test scores not accounted for by gaze patterns.

With the current study, we aimed to advance the understanding of intelligence and personality by additionally considering gaze information captured in the test situation. In a sample of 182 adults^27^, we combined assessments of Big Five personality traits and fluid intelligence test scores (based on subtests from the Culture-Fair IQ Test; CFT 20-R) with eye-tracking information recorded while participants completed the CFT 20-R. All research questions were investigated with machine learning models.

First, how are Big Five personality traits associated with fluid intelligence test performance (Research Question 1; RQ1)? We hypothesized that we would find significant associations with Openness to Experience (positive) and Neuroticism (negative). As prior research has documented inconsistent and mostly negligible correlations^21^, we did not expect significant relationships between the other three Big Five dimensions and fluid intelligence test performance.

Second, do gaze patterns predict intelligence test performance (Research Question 2; RQ2)? We hypothesized that gaze patterns would explain a substantial amount of variance in intelligence test performance on the speeded test used in our study.

Third, we linked gaze information and personality traits: How are personality traits related to gaze patterns, and particularly, to gaze patterns indicative of higher intelligence test performance (Research Question 3; RQ3)? We hypothesized that we would find positive (Openness to Experience) and negative (Neuroticism) associations, mirroring the pattern for personality–intelligence associations (see RQ1). We assessed the other Big Five personality traits in an exploratory fashion without specifying concrete hypotheses.

For RQ2 and RQ3, we asked whether gaze information is associated with intelligence test performance and personality without further characterizing the gaze patterns (i.e., without identifying strategies). Hence, to follow up on RQ2 and RQ3, another relevant question is what are the specific characteristics of gaze patterns that indicate higher intelligence test performance (Research Question 4; RQ4)? It seems likely that such gaze patterns would resemble the constructive matching strategy; however, given the time limit on the test in our study, strategies such as constructive matching and response elimination might not be as identifiable in our data in comparison with previous studies involving untimed tests.

Fifth, to what extent do personality traits predict intelligence test performance above and beyond the amount of variance explained by gaze patterns (Research Question 5; RQ5)? We expected that personality traits (especially Openness to Experience and Neuroticism) would still uniquely contribute to the prediction of intelligence test scores when the amount of variance explained by gaze patterns was accounted for.

We revisited all research questions involving personality using personality facets.

## Results

Table 1 presents descriptive statistics and bivariate correlations. Regarding the intelligence test, we used the overall score, which includes the scores from the considered subtests (i.e., CFT 20-R Blocks 1, 3, and 4) in the analyses (see the Measures section). Supplementary Table 1 presents correlations for personality facets.

**Table 1:** Descriptive statistics and bivariate correlations for all variables

	Descriptives		Bivariate correlations							
	*M*	*SD*	1	2	3	4	5	6	7	8
Big Five										
[1] O	2.69	0.35								
[2] C	2.47	0.46	−0.18*							
[3] E	2.37	0.45	0.21**	0.21**						
[4] A	2.51	0.40	0.26***	0.01	0.14					
[5] N	1.89	0.56	0.12	−0.36***	−0.28***	−0.02				
CFT 20-R										
[6] Block 1	13.10	1.64	0.13	−0.03	0.06	0.17*	0.00			
[7] Block 3	11.20	1.70	0.16*	0.00	0.01	0.11	−0.01	0.41***		
[8] Block 4	6.41	1.73	0.22**	−0.16*	0.02	0.04	−0.04	0.27***	0.32***	
[9] Score	30.66	3.81	0.23**	−0.08	0.04	0.14	−0.02	0.74***	0.77***	0.72***

*N* = 182, *O* = Openness to Experience, *C* = Conscientiousness, *E* = Extraversion, *A* = Agreeableness, *N* = Neuroticism, Block 1 = Series continuation, Block 3 = Matrices, Block 4 = Topological conclusions, Score = CFT 20-R overall score composed of scores from Blocks 1, 3, and 4.

*p < 0.05, **p < 0.01, ***p < 0.001.

### Predicting CFT scores from personality (RQ1)

We relied on a machine learning model (elastic net) that used personality traits as predictors of CFT scores. The results were obtained with 10-fold cross-validation to ensure robustness. Specifically, the data set was divided into 10 equally sized parts (called folds), the model was trained on nine parts and then evaluated on the withheld part. This process was repeated for all 10 parts, and subsequently, the results were averaged.

**Fig. 1:**
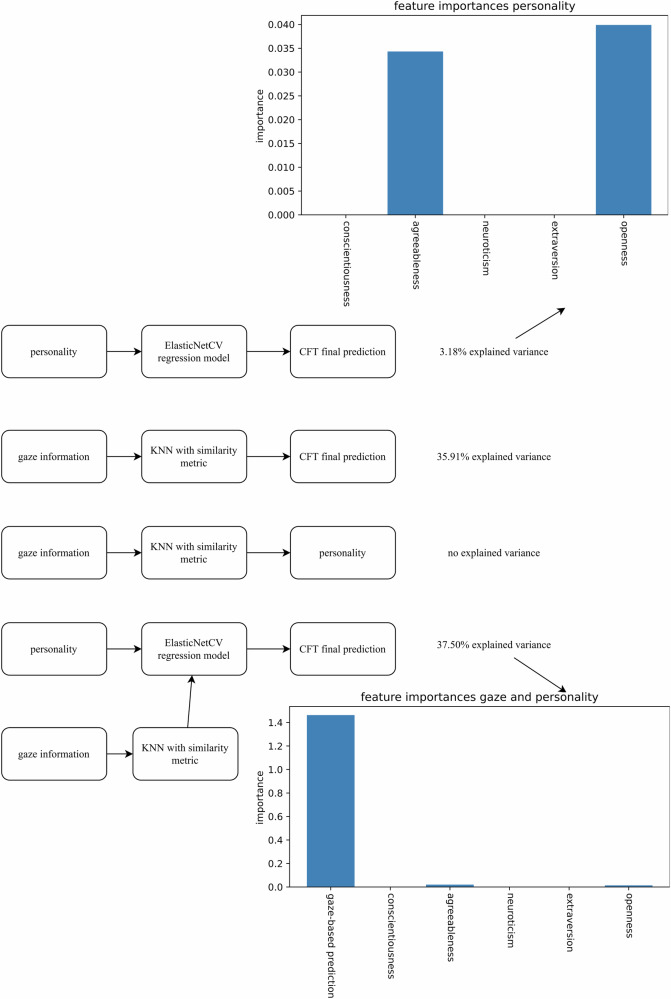
Overview of the results for research questions 1, 2, 3, and 5. The results for RQ1 (predicting CFT scores from the Big Five personality dimensions), RQ2 (predicting CFT scores from gaze information), RQ3 (predicting personality from gaze information), and RQ5 (predicting CFT scores from both gaze information and the Big Five personality dimensions).

The model explained 3.18% of the variance in CFT scores based purely on the Big Five personality traits. An analysis of feature weights, which can be interpreted relative to each other and are similar to absolute values of regression coefficients, showed that Openness to Experience and Agreeableness were the only two personality traits that meaningfully contributed to the model’s success, with coefficients (i.e., feature weights) of 0.03989 and 0.03431, respectively (see Fig. 1). As a robustness check, we reran the analyses using a “standard” multiple regression. The results revealed that only Openness to Experience and Agreeableness were statistically significant predictors of intelligence test performance (see Supplementary Table 2). Because several participants had to be excluded from our main analyses due to problems with their eye-tracking data (see the Sample section for details), we performed an additional robustness check. Specifically, we conducted our machine learning analyses for RQ1 on the whole sample without data exclusions. These analyses revealed the same pattern of findings in that meaningful predictive contributions were restricted to Openness to Experience and Agreeableness (see Supplementary Table 3).

We then set up the same machine learning model as in the analyses for the Big Five personality traits but with the personality facets as features. A total of 7.67% of variance in CFT scores could be explained. Facets with nonzero coefficients were the Openness facets Ideas (0.0034), Values (0.0028), and Fantasy (0.0013); the Neuroticism facet Self-consciousness (0.0019); the Extraversion facet Assertiveness (0.0013); and the Agreeableness facets Trust (0.0027) and Compliance (0.0027).

### Predicting CFT scores from gaze patterns (RQ2)

For RQ2 (and the subsequent research questions), eye-tracking data were additionally considered. For the eye-tracking data, we first divided the stimulus (i.e., each item from the CFT 20-R subtests) into areas of interest that corresponded to their semantic function, meaning that we focused on questions, targets, distractors, and others (including the clock and progress bar). Then, scanpath (i.e., gaze sequence) similarity was defined, providing information about whether participants with similar gaze behavior had similar CFT scores. The similarity between scanpaths was used to make predictions for CFT scores (see the Method section for technical details). Applying leave-one-participant-out cross-validation to ensure reliable and robust results that did not overfit our model to this specific data set, the method was able to explain 35.91% of the variance in CFT scores in our data set, indicating that the gaze data provided substantial information about participants’ success on an intelligence test. The first six to eight items in each block could be predicted almost perfectly, but as virtually all participants solved these items correctly, they explained no variance in the CFT scores. Consequently, gaze patterns for these items carried little information about participants’ scores, whereas the following items were much more informative.

### Predicting personality traits from the pairwise similarity of scanpaths (RQ3)

**Fig. 2:**
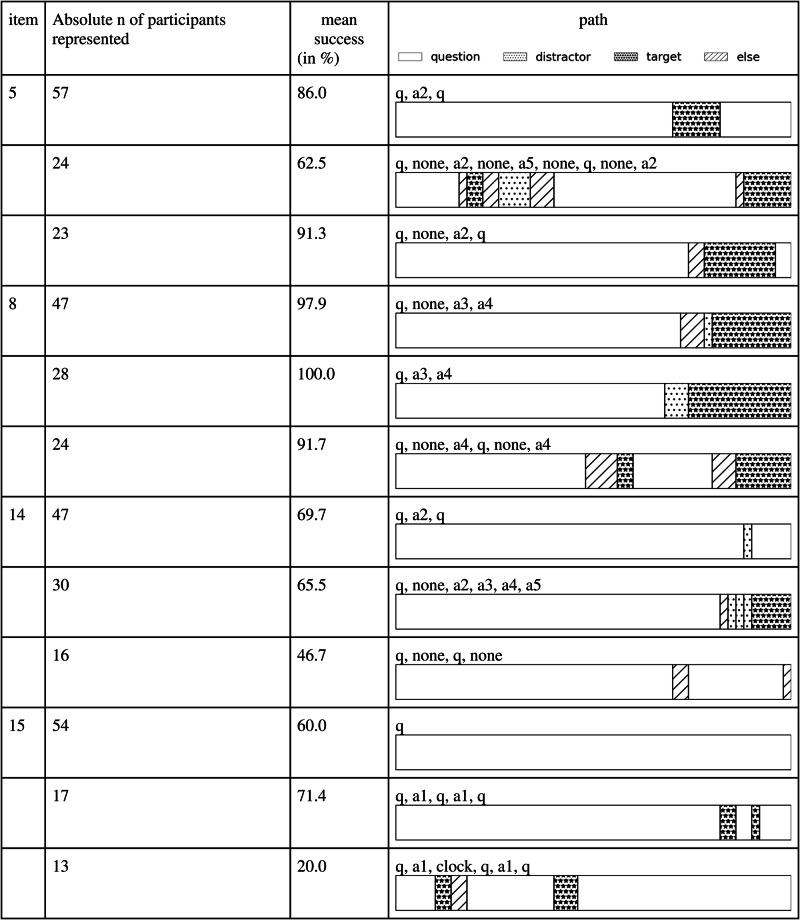
Overview of gaze patterns for Block 1 (series continuation). Gaze patterns for items for which gaze-based predictions were significantly above chance. Note. q question; a answer (response options 1–4).

The same leave-one-participant-out cross-validation approach as for the prediction of CFT scores was employed to predict personality traits. However, the model explained no variance in the personality traits, indicating that the personality traits were not significantly linked to gaze patterns while participants solved intelligence test items. The model that used only items for which gaze similarity produced CFT predictions that were significantly above chance (i.e., gaze patterns indicative of higher intelligence test performance) yielded the same results (see Fig. 2). In additional analyses using the facets, the results indicated that such a model could only explain more than 1% of the variance in four facets: Self-Consciousness (Neuroticism facet), Depression (Neuroticism facet), Assertiveness (Extraversion facet), and Competence (Conscientiousness facet). The explained variance was 1.14%, 1.84%, 2.41%, and 1.16%, respectively. Focusing on gaze patterns indicative of higher intelligence test performance, we identified four facets for which more than 1% of the variance could be explained: Assertiveness (4.27%), Self-Consciousness (1.10%), Aesthetics (Openness facet, 1.79%), and Fantasy (Openness facet, 1.76%).

### Extracting strategies (RQ4)

**Fig. 3:**
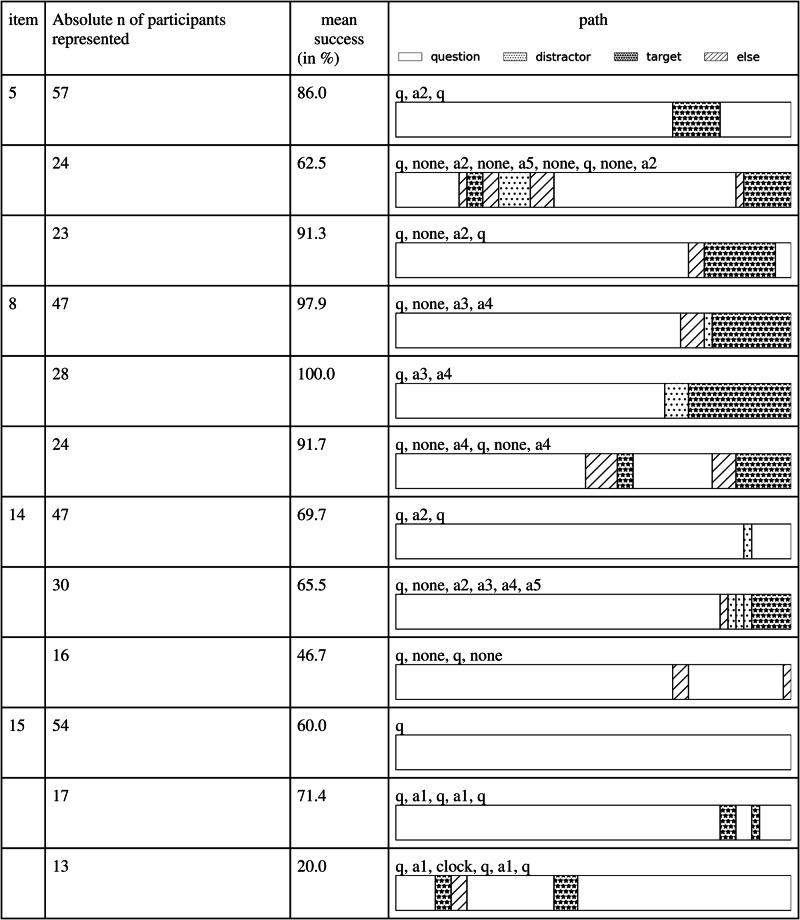
Overview of gaze patterns for Block 3 (matrices). Gaze patterns for items for which gaze-based predictions were significantly above chance. Note. q question; a answer (response options 1–4).

**Fig. 4:**
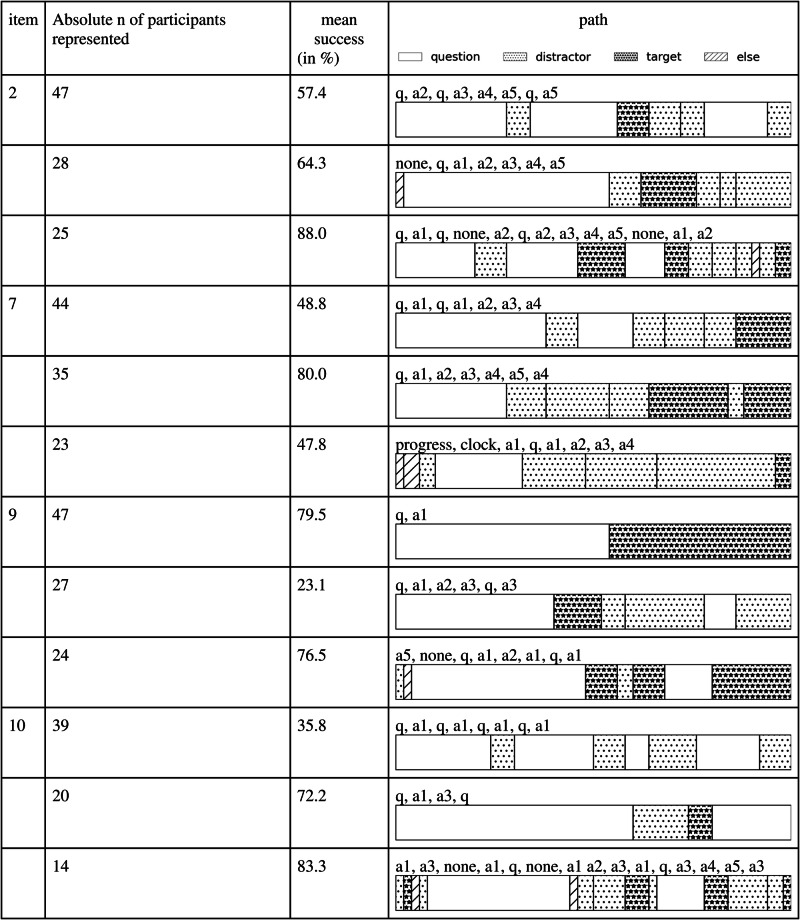
Overview of gaze patterns for Block 4 (topological conclusions). Gaze patterns for items for which gaze-based predictions were significantly above chance. Note. q question; a answer (response options 1–4).

Next, we attempted to gain a better understanding of characteristics of gaze patterns by focusing on test-solving strategies. We had initially planned to label representatives of common intelligence-test-solving strategies (constructive matching and response elimination) by hand and use them as a foundation for the clustering or classification of those strategies. However, it was difficult to find sufficiently clear examples for both strategies with the data for each item, possibly due to the timed nature of the test. Furthermore, declaring that specific gaze patterns represent certain strategies would have introduced bias if they did not match that strategy exactly. Alternatively, we applied an iterative method to investigate the most representative scanpaths for each question on the test (see the Method section for technical details). Figures 2, 3, and 4 show the most representative scanpaths for all questions where predictions of test scores were significantly above chance (indicating that strategy use was indicative of participants’ success) for items from the CFT 20-R Blocks 1 (series continuation), 3 (matrices), and 4 (topological conclusions), respectively. In addition to the most representative scanpaths, we calculated mean success rates for scanpaths that were similar to the representative one, as well as how many scanpaths for that item listed the given scanpath as one of their most similar scanpaths. This approach provided insights into which strategies participants used and how many participants employed a similar strategy.

To illustrate the use of strategies, we present Item 9 from the first block (series continuation) as an example (see Fig. 2). The scanpath that was most representative of this item was referenced by 57 participants and offered a 91.1% success rate on average. It was characterized by fixations on the question, followed by fixations on all five response options successively, before finally reverting to Option d, the target. This behavior suggested that participants were looking for an option with specific characteristics, and once that option was identified, there was only a short fixation on Option e before they settled for Option d (i.e., the target). This approach resembles the constructive matching strategy^8,29,30^. The second scanpath had many transitions between the question and the response options, as well as successive fixations on different options. It was referenced by 36 scanpaths, and 66.7% of these scanpaths belonged to successful participants. Scanpath 3 was very similar to the second one, with slightly different fixations on options. A total of 17 scanpaths were similar to this one, and 70.6% of them belonged to participants who solved this task correctly. The two approaches are very similar and are more aligned with the response elimination strategy^30,37^.

In the first block (series continuation), gaze patterns that are more akin to constructive matching usually led to a high chance of solving the corresponding item correctly. This point is illustrated, for example, by scanpaths that are variations of the sequence “question → distractor → target” or “question → target.” On the other hand, scanpaths that had more transitions to and from the “question” area of interest of an item often had success rates of less than 50% and appeared more similar to response elimination. The third block (matrices) showed similar patterns, but they were less pronounced. Finally, the fourth block (topological conclusions) as a whole was characterized by relatively more transitions, leading to less pronounced differences in scanpaths. There seemed to be a pattern where more transitions to and from the “question” area of interest indicated less success. Furthermore, relatively longer first fixations on the question seemed to be an indicator of a more successful strategy. Cumulatively, the overview of gaze patterns in Figs. 2, 3, and 4 suggest that gaze patterns resembling response elimination were less successful than gaze patterns that were more aligned with constructive matching.

To better contextualize the findings, we also considered that the three blocks included in our analysis covered different types of tasks, which could result in different scanpaths. For instance, the initial time participants spent looking at the question varied dramatically between blocks. Block 3 (matrices) had the longest looking periods, whereas Block 4 (topological conclusions) had the shortest, indicating that participants’ approach to these tasks was inherently different. The completion of matrix tasks requires participants to understand the patterns in the matrix, so participants are expected to spend a lot of time looking at the matrix. Gaze behavior for Block 3 showed the least variety with respect to response options, and oftentimes only a few options were considered. On the other hand, for topological conclusions, only a few fixations on the question are required to grasp what to look for, but scanning the five options for matching topography is time-consuming. Block 1 (series continuation) is somewhere between these two extremes. Furthermore, participants seemed to consider more options and spend more time looking at different options in Block 4 (topological conclusions) compared with the other two subtests. Gaze patterns for Block 1 (series continuation) were similar to Block 4 but not as pronounced. In Block 1, overall, a greater emphasis was put on the question, and there was less variety in the response options. The inherent difficulty in solving an item also varied from block to block. Block 4 (topological conclusions) in particular appeared to be more challenging for participants, as the success rate for this block was lower than in Blocks 1 and 3. The higher difficulty of Block 4 was reflected in gaze behavior, too, as scanpaths in Block 4 have a lot more transitions overall, which may be interpreted as a sign of confusion and could indicate a fall-back to response elimination.

### Predicting CFT scores by combining gaze information and personality traits (RQ5)

By using a combined feature vector consisting of gaze-based predictions and personality traits, the resulting elastic net explained 37.50% of the variance. These results were obtained with 10-fold cross-validation. Looking at feature weights (see Fig. 1) revealed that gaze information (as encoded in our CFT prediction based on gaze data) had a coefficient of 1.416, whereas Openness and Agreeableness had coefficients of 0.018 and 0.019, respectively. These feature weights show that the majority of the variance was explained by gaze behavior; however, personality traits also carried some information and significantly predicted intelligence test performance above and beyond the variance explained by gaze patterns.

When combining gaze-based predictions with personality facets, our model was able to explain 38.02% of the variance. Gaze information had a feature weight of 1.288, whereas the Ideas facet of Openness had a feature weight of 0.0022, the Values facet of Openness had a feature weight of 0.0001, the Trust facet of Agreeableness had a feature weight of 0.0004, and the Compliance facet of Agreeableness had a feature weight of 0.0015. Again, these weights indicate that gaze explained the most variance, but personality facets provided complementary information about participants’ intelligence test scores.

As a final robustness check, we reran all our analyses with a random training-test split of 90–10 and repeated the process 1000 times. Except for minor details (90–10 instead of 80–20 and 1000 repetitions instead of 100), we used the protocol that Jankowsky et al. used in a recent study^38^. Differences in the amount of variance explained between the model based on Jankowsky et al.’s protocol and our original model reported in the manuscript ranged from 0.00 to 0.01 for all research questions.

## Discussion

This study combined eye-tracking information with intelligence test scores (CFT 20-R) and personality (Big Five) self-report measures and leveraged the potential of machine learning to answer a set of research questions. Machine learning (elastic net) results revealed that personality was able to explain a small amount of variance (3.18%) in intelligence test scores. Openness to Experience and, surprisingly, Agreeableness contributed the most to the prediction, with feature weights of similar size. Agreeableness includes components such as compliance, trust, and tender-mindedness. A previous study that obtained a significant positive association between Agreeableness and (fluid) intelligence suggested that the cooperative attitudes of participants scoring high on Agreeableness could be the shared underlying factor in the relationship with intelligence^39^, as cooperative social interactions are a major force in the development of cognitive skills^40^. Considering that our data stemmed from a larger study including multiple tests and questionnaires, it may further be the case that more agreeable individuals—who were presumably more cooperative in the test situation itself and more likely to comply with instructions—were more focused on the intelligence test and experienced more positive thoughts and emotions about donating their time toward contributing to scientific knowledge, which helped them achieve higher test scores^41^. Further, a model based on Big Five facets explained a larger amount of variance in intelligence test scores (7.67%). The largest coefficient was obtained for the Ideas facet of Openness, followed by the Values facet of Openness, and the two Agreeableness facets Compliance and Trust. Self-Consciousness (Neuroticism), Assertiveness (Extraversion), and Fantasy (Openness) also contributed to the prediction. The finding for the Ideas facet (Openness), which involves intellectual curiosity, squares well with prior research identifying Ideas as the Big Five facet that is most strongly related to fluid intelligence^21,42^. The facet-level analyses also cast new light on the finding for Agreeableness, as being compliant and cooperative and believing that others are generally trustworthy and well-intentioned seemed to have been relevant to test performance in the context of our study. Also, whereas we did not obtain effects of Neuroticism (contradicting our hypothesis) and Extraversion (no hypothesis specified) on the trait level, one facet from each trait still made contributions to the prediction. In general, these findings underscore the value of facet-level analyses, which allowed us to dig deeper into the details of personality–intelligence associations.

The most important aspect of our work, however, lies in how we combined behavioral data with “traditional” psychological measures (intelligence, Big Five personality). The model set up to predict intelligence test scores using gaze patterns showed that a substantial amount of variance in intelligence test performance was explained by gaze patterns (35.91%). Our study thus adds another piece of evidence that underlines the importance of eye movements during the test for explaining intelligence test performance. For instance, gaze information previously explained 45% of the variance in test scores on the Wiener Matrizen-Test 2 (WMT-2)^43^, a matrix-based fluid intelligence test^37^. Another study focusing on “gains” in fluid intelligence test performance on Raven’s Advanced Progressive Matrices (APM)^44^ revealed that about one third of the variance in score gains could be attributed to characteristic changes in eye-fixation patterns^5^. Of course, these previous studies differed from our work in, for example, their measures, exact research questions, and analytical approaches, and, furthermore, they relied on unspeeded intelligence test. All these factors prevent a direct comparison with our work. Still, our study contributes to the existing body of work focusing on participants’ eye movements when taking intelligence tests, while expanding the scope of this literature due to the speeded nature of our test.

Third, gaze patterns did not explain any variance in personality traits, indicating that gaze patterns were not “personality loaded.” This finding applied to both intelligence-test-performance-relevant gaze (i.e., when we used only items for which gaze similarity produced CFT predictions that were significantly above chance) and gaze patterns in general. Hence, there was no support for our hypotheses that Openness to Experience and Neuroticism would be linked to gaze information. Nevertheless, the results from the facet-level analyses revealed that individual differences in Self-Consciousness and Depression (Neuroticism facets), Competence (Conscientiousness facet) and Assertiveness (Extraversion facet) systematically explained variation in general gaze patterns. For example, Self-consciousness is linked to social anxiety and avoidance behaviors and has already been found to be related to gaze patterns and other behavioral measures in prior work^45,46^. By contrast, Assertiveness describes the tendency to be dominant and take charge^15^, and our study’s findings suggest that Assertiveness tendencies were also reflected, to some extent, in gaze patterns. Moreover, Assertiveness, Self-Consciousness, and Aesthetics and Fantasy (Openness facets) systematically explained variance in intelligence-test-performance-relevant gaze. Interestingly, the Openness facet Ideas, which carried the most weight in the prediction of intelligence test scores in the facet-level analyses, did not play a role in the gaze patterns that were conducive to intelligence test performance. Whereas the Ideas facet of Openness (and not Aesthetics) is typically related to fluid intelligence test performance^21,42^, our results revealed that it was actually the Aesthetics facet that added to the prediction of gaze. A previous study compared predictive effects of intellectual curiosity (akin to Ideas) and a broader Openness trait including Aesthetics on knowledge attainment. This study found that Openness (and not intellectual curiosity) benefitted learning^47^. Benefits of Openness (including Aesthetics) may have emerged because Openness predisposes individuals to perceiving and extracting information across situations, including day-to-day experiences but also more cognitively challenging undertakings^47^. We propose that a similar argument focusing specifically on the part of Openness that engages with Aesthetics could be made for our study. To the best of our knowledge, no study has yet adopted eye-tracking to examine whether and how Big Five personality traits and facets translate into gaze patterns while participants complete intelligence test items. Our findings thus expand on previous work on personality and intelligence tests without eye-tracking^30^ as well as eye-tracking studies on personality without a focus on intelligence-test-related gaze patterns^48–50^. However, when interpreting our findings, readers should remember that our study focused on gaze during a very specific task in a specific test situation in the laboratory. This specificity may have limited the impact of personality traits on such gaze patterns, as compared with studies on gaze patterns and personality conducted in real-world and dynamic contexts, which leave more room for personality to be expressed^51^. It is also conceivable that personality traits may have played a more important role in other occasions, such as longer testing sessions in which personality could have “taken over” to a greater extent, or in high-stakes assessment situations. In addition, certain personality traits (e.g., Openness) can partially compensate for cognitive decline in old age^52^, and we may have found more personality-loaded gaze patterns in samples with on average older adults. It may also be the case that personality traits that capture typical behavioral tendencies could have a larger impact on gaze patterns in unspeeded intelligence tests, as the time limit may have suppressed some of these typical behavioral tendencies in individuals’ looking behavior.

Fourth, we sought to identify strategies on the basis of gaze information, thus shedding light on the “black box” of gaze patterns. There were indications that gaze patterns reflected test-solving strategies from the literature in terms of constructive matching and response elimination^8,29,30,37^ to some extent. In addition, the gaze patterns that were more closely aligned with constructive matching (response elimination) seemed to be more (less) successful. We suspect that because the intelligence test used in our study was implemented with a time limit, strategies may have been more blurred than in previous studies. Specifically, in several instances, gaze patterns could not be clearly mapped onto one strategy and were located somewhere between constructive matching and response elimination. Nevertheless, there is a need for detailed analyses of the processes that take place during intelligence testing^53^, and our study makes a relevant contribution to this line of research.

Fifth, the results revealed that a combined feature vector consisting of gaze-based predictions and personality traits explained about 37.50% of the variance in intelligence test performance. As hypothesized, personality traits made a significant (but very small) contribution above and beyond the effects of gaze patterns. These findings underscored that both gaze patterns and (to a lesser degree) personality (Agreeableness, Openness to Experience) were associated with intelligence test performance. Whereas the analyses for RQ3 described above found that gaze information and personality traits were unrelated, the results from the model combining personality and gaze patterns suggested that gaze and personality were independent predictors of intelligence test performance. The results from facet-level analyses complemented these insights and showed that mostly gaze, but also specific Openness and Agreeableness facets (Ideas > Compliance > Trust > Values) predicted intelligence test performance (overall explained variance: 38.02%). It is noteworthy that when predicting intelligence test scores from personality and gaze, the difference in explained variance between the model including traits versus facets was minimal, whereas in the models that used only personality traits (or facets) to predict intelligence test scores, the facet model explained almost 2.5 times more variance than the trait model.

Overall, it must be acknowledged that behavioral data strongly outperformed “traditional” questionnaire data (personality traits and facets) in predicting intelligence test performance. This finding has implications for psychological assessment in general, as it underlines the importance of actual behaviors that have been widely neglected in research combining personality and intelligence. In fact, research on intelligence and personality, on the one hand, and research on intelligence test performance and eye-tracking, on the other hand, have developed in complete isolation from each other. It is a major contribution of this study that it brings the two lines of research together to reveal insights into the relative predictive strengths of personality and gaze patterns. At the same time, a more differentiated interpretation of the obtained pattern of results that takes the distinct types of measures into account is warranted. For instance, behavioral measures are objective indicators, whereas self-reports represent subjective judgments. Further, behavioral measures tap state-like responses in specific and highly structured situations, whereas self-report measures ask individuals to reflect on their behaviors across a range of unstructured real-life situations^54^. State measures are typically more strongly related to situation-specific performance than trait measures (even trait measures of the same construct^55^). We thus caution that the discussion of our findings should not lead to the conclusion that “traditional” questionnaire data should be discharged in research on intelligence test performance. Instead, to build a stronger science of the interface between intelligence, personality (and noncognitive constructs more broadly), and human behavior, we need to think more clearly about the meaning and interplay among constructs located at different levels of granularity and time scales, the proximity of a single construct to what we consider to be the central “outcome” variable of interest, and the centrality of different aspects within this overall network, as well as the impact of different assessment strategies.

Several limitations of the current study should be noted. We have argued that some patterns of findings may have emerged because the intelligence test in our study was timed. However, we did not conduct systematic comparisons between speeded and unspeeded versions of the test (or parts of the same test). Further, eye-tracking information was obtained in one session, and it would have been interesting to explore day-to-day shifts in gaze patterns, cognitive performance, and personality (see, e.g., ref. ^23^, for such a study that did not include eye-tracking). Next, our sample comprised adults with a university entrance exam (most of them university students). Our sample was not a random sample, nor was it population-representative, and these limitations should be kept in mind when interpreting our findings. Future research would also do well to include further personality traits and frameworks (e.g., HEXACO personality^21,56^). In addition, a comprehensive 70-facet measure has recently been developed^57^, and exploring links between these facets and gaze patterns could provide important further insights.

Furthermore, although the facet-level analyses revealed interesting insights, some caution is warranted due to the combination of having a relatively modest sample size relative to the analytic complexity of the models, the fact that we found effects that were not necessarily in line with the hypotheses, and the possibility that examining a multitude of facets increases the risk of Type I errors. Hence, replications are needed before more definite conclusions on the link between specific facets and gaze patterns can be made.

Even though our analyses were well-suited for addressing the research questions, future research could expand on them, for example, by maximizing performance prediction by using a black box–machine learning model with post hoc explanations or by paying closer attention to the sequential and temporal structure of the eye-tracking data (e.g., by identifying “moments of confusion” while participants work on the test^58^). Another important avenue for future research centers on investigating which gaze behaviors show trait-like patterns. Specifically, are certain individuals more likely to show a particular type of gaze pattern consistently across items? Similarly, are particular types of items likely to elicit particular gaze patterns? Relatedly, including other trait measures beyond Big Five personality traits (e.g., Need for Cognition, specific motivational constructs^30^) as predictors of gaze patterns could reveal relevant insights.

In a study by Lynn et al.^59^ on Raven matrices, a three-factor structure was identified (Gestalt continuation, verbal reasoning, and visuospatial ability). A more differentiated understanding could thus be achieved by first conducting a factor analysis of intelligence test items and then regressing different gaze strategies on the factors. Eye-tracking data could also be fruitfully complemented by other behavioral data to gain an even better understanding of how individuals take intelligence tests. For instance, a recent study focused on visual exploration by using the computer mouse to unveil strategies in matrix reasoning tasks^60^, and mouse movements have also been linked to Big Five personality traits^61^. In addition, the reliance on physiological measures, such as heart rate, skin conductance, or cortisol levels^62^, could reveal further relevant insights. The use of brain imaging to assess cognitive processes while individuals work on an intelligence test^63^ offers additional interesting possibilities. The study of brain-to-brain synchronization, which nowadays often relies on the use of portable EEGs in real-life learning settings^64,65^, could be another promising avenue. Specifically, we suggest that the brain waves of those with higher intelligence test performance may be more “in sync” and that the use of similar strategies (as manifested in similar gaze patterns) could be the driving force behind such brain-to-brain synchronization patterns.

Also, the test we used in our study was a measure of fluid intelligence, and it remains to be explored whether our results can be transferred to other intelligence test measures. For instance, scores on crystallized intelligence measures are typically more strongly related to Openness to Experience than fluid intelligence test scores are^21^, and it may also be easier to identify gaze patterns linked to Openness when using such tests. We further suggest that for other types of intelligence tests with formats that allow reliable tracking of the “knowing beforehand”-component inherent in constructive matching strategies, similar eye-tracking results as in our and related previous work^28,30,32^ may be obtained. However, eye-tracking research so far has strongly relied on figural fluid intelligence test content, and future research is needed to expand the scope. Future research could also use intelligence tests that better correspond to the CHC model of intelligence (e.g., Woodcock–Johnson IV) than the CFT 20-R, which would allow to examine the relationships of eye-gaze patterns to the different components of intelligence.

Finally, in addition to implications for research, what implications for practice arise from our findings? Strategies that individuals use to successfully solve fluid intelligence tests have already informed attempts to raise test scores by training such strategies. Our findings also suggest that training individuals to adopt constructive matching strategies may, within everyone’s cognitive boundaries, have some effects (but see, e.g., ref. ^5^, for a critical discussion). At the same time, our findings indicate that strategy training, at least based on commonly extracted strategies such as constructive matching, is probably much less helpful for speeded tests than for unspeeded tests.

To conclude, the present study bridged research on intelligence and personality with the behavioral-process-related lens provided by eye-tracking. We showed that personality (Agreeableness and Openness to Experience) and gaze patterns predicted intelligence test performance, with gaze patterns explaining more than 10 times as much variance as personality traits. Facet-level predictions of intelligence test scores explained a larger amount of variance than trait-level predictions, with the largest coefficients obtained for Ideas and Values (Openness) and Compliance and Trust (Agreeableness). Personality traits were not linked to gaze patterns, but personality facets (especially Self-Consciousness from Neuroticism and Assertiveness from Extraversion) were. To some extent, gaze patterns resembled strategies previously identified in the literature in terms of constructive matching and response elimination; however, the use of a speeded test likely contributed to the fact that, in our study, test-solving patterns were more blurred than in previous studies and still remain, to some extent, a black box to be further unpacked in future research. We also found that personality (both traits and facets) explained a small amount of variance above and beyond the variance explained by gaze patterns. Whereas behavioral data thus clearly outperformed “traditional” psychological measures (Big Five personality) in predicting intelligence test performance, our results also underline the independent contributions of personality and gaze patterns in predicting intelligence test performance.

## Methods

### Participants and procedure

We used the publicly available TüEyeQ data set^27^, which is part of a larger scale lab study on self-regulation (see ref. ^27^, for details; see also ref. ^66^). The TüEyeQ data set contains eye movement data from 315 university students while they solved the first part of the CFT 20-R, an intelligence test employing figural tasks^67^, and sociodemographic data. We further enriched this data set with information on the Big Five (available upon request from the TüEyeQ project team). All data were collected throughout 2018, beginning in February and finishing in December, in a sample of adults with a university entrance qualification, no preexisting neurological or psychiatric conditions, and no visual impairment above 3 diopters. After excluding 86 participants’ eye-tracking data for various reasons (58 because of a poor tracking rate [below 80%], 11 due to errors during the presentation of our stimulus, and 17 due to incomplete data), data from 229 participants remained. As the eye movements of several participants did not meet the accuracy standards required for our analyses, 43 more participants were excluded after manual inspection (e.g., if it was obvious that a participant’s gaze did not match the stimulus at all, indicating movement after calibration that tends to lead to large offsets in x and y coordinates). These 43 participants showed substantial offsets in their fixation location, likely caused by head movements after calibration, and rendering a reliable matching of fixations to areas of interest impossible. In addition, another four participants were not considered because their data from different tasks could not be matched reliably, or their eye-tracking data could not be paired with participants’ intelligence test performance data. The final sample consisted of 182 participants (*M*_age_ = 23.32, *SD*_age_ = 2.89; 71.82% women) for our analysis (see also ref. ^27^). A total of 95.58% were native German speakers. The most frequently reported primary subjects of study were social sciences, journalism, and information (25.27%) and arts and humanities (23.63%), followed by education (15.38%), and natural sciences, mathematics, and statistics (11.54%). With respect to family background characteristics, 44.51% of the participants’ mothers and 51.10% of the participants’ fathers had a university degree. Supplementary Table 4 provides additional demographic information.

All tests and questionnaires were administered on a 17-inch laptop with a resolution of 1920 × 1080. The eye trackers were SMIred manufactured by SMI and run at 250 Hz. Calibration was performed with a 9-point calibration before each test and questionnaire, and lighting conditions were kept constant throughout each recording session and across sessions. Participants could choose whether they wanted to use a mouse or the built-in touchpad for their interactions with the laptop.

### Inclusion and ethics statement

This research was conducted in Germany. The Ethics Committee at the Psychological Institute at the University of Tübingen confirmed that the procedures fulfilled all ethical standards of research with human subjects. All participants were informed about the study in writing, and they gave consent for their anonymous data to be analyzed and published. Because we used self-constructed pseudonyms, participants had the option to revoke this consent at any time (see also ref. ^27^).

### Measures

Established instruments were used to assess intelligence and personality. Gaze patterns are described in the Results section and not covered in the Method section.

#### Intelligence

Intelligence was measured with the first part of the CFT 20-R^67^. The CFT 20-R builds on Cattell’s culture fair intelligence test^68^, which was developed as a language-free measure of fluid intelligence. The CFT 20-R contains four tasks for assessing fluid intelligence: series continuation, classification, matrices, and topological conclusions. Each task was presented in a block, prefaced by an explanation and an example with a solution. In contrast to the task itself, these introductions did not have a time limit. The blocks themselves had time limits of 3 and 4 min, respectively, as suggested by the test manual. We restricted our analysis to Blocks 1, 3, and 4 of the CFT 20-R (i.e., series continuation, matrices, and topological conclusions) and excluded Block 2 (classification) from our analysis. The layout of Block 2 had different options grouped tightly together; therefore, the accuracy of the eye trackers was not sufficient to reliably distinguish between them. All of the tasks required participants to choose the correct answer from five possible options. Correct responses were scored 1, and incorrect responses were scored 0. Block 1 (series continuation) and Block 3 (matrices) consisted of 15 items each, thus each yielding a maximum score of 15. Block 4 (topological conclusions) consisted of 11 items (maximum score of 11). Consequently, the overall maximum score for the blocks considered in this study was 41.

In the series continuation task, participants were presented with a series of three figures and asked to choose which figure out of five alternatives best continued the progressive series. In the matrix task, participants were asked to identify the underlying pattern of figures presented in a matrix and then to select a suitable figure to complete the matrix. Lastly, in the topological conclusions task, participants were provided with a reference figure containing one or more dots. Their objective was to comprehend the topological connections between the dots and the overall figure and to subsequently select a figure where a dot could be positioned with the same topological relationship^67^.

The CFT 20-R subtests can be integrated into the Cattell–Horn–Carroll framework (CHC model), which is the most prevalent intelligence model today^69^. In the CHC model, a general intelligence factor, *g*, resides at the apex of the hierarchy, with up to 16 factors representing broad cognitive abilities (e.g., fluid intelligence, crystallized intelligence, short-term memory, visual processing) grouped underneath g and up to 80 factors representing narrow cognitive abilities that further differentiate the broad abilities grouped underneath the broad cognitive abilities. The CFT 20-R subtests used in our study map onto the fluid intelligence factor at the level of broad cognitive abilities in the CHC model and onto induction and general sequential reasoning on the level of narrow abilities^70^.

#### Big five personality traits and facets

The Big Five were assessed with the German version of the Revised NEO Personality Inventory (NEO PI-R^71^). Strictly, the NEO-PIR is a measure which corresponds to the Five Factor Model (FFM) and not the Big Five. To date, however, the models are considered to be so closely congruent that the FFM and Big Five are often referred to interchangeably^72^. The questionnaire consists of 240 items that were answered on a 5-point Likert scale (ranging from 1 = strongly disagree to 5 = strongly agree). Sample items for each scale are “I work hard to accomplish my goals” (Conscientiousness), “I go out of my way to help others if I can” (Agreeableness), “I am easily frightened” (Neuroticism), “I really like most people I meet” (Extraversion), and “I have a wide range of intellectual interests” (Openness to Experience). Internal consistency estimates (Cronbach’s alpha) for all scales were good and ranged from *α* = .85 (Openness) to *α* = .94 (Neuroticism). In additional analyses, we relied on the Big Five facets. For the 30 facets, internal consistency estimates (Cronbach’s alpha) ranged from *α* = .63 for Values (Openness) to *α* = .90 for Depression (Neuroticism).

### Analytic approach

Our analyses consisted of multiple steps that built on each other and served to answer specific research questions. As the analyses we applied (machine learning, analyses of eye-tracking data) are not standard in many research areas, we describe our analytical approach in detail, organized along the core research questions.

#### Predicting CFT scores from personality (RQ1)

To address RQ1 and to keep the analyses consistent with the subsequently performed machine-learning-based analyses of gaze patterns, we first used a machine learning model to predict scores on the intelligence test from personality traits. The machine learning approach allowed us to go beyond the conventional statistical models that are typically employed in research on intelligence–personality associations. Machine learning makes it possible to capitalize on more complex models with a focus on predictions rather than explanations of collected data. We thereby aimed for conservative metrics of model fit and a good level of generalizability by using parameter-regularization and cross-validation. Regularization adds a penalty to the optimization function that penalizes high-parameter coefficients and thereby helps to find a simple solution that still fits the given data. Furthermore, cross-validation was used to ensure robust results that did not overfit the model. Cross-validation describes the practice of partitioning the data set into *n* equally sized parts (called folds; common choices for *n* are 5 or 10), training the model on *n*-1 parts, and evaluating it with the part that had been withheld. Repeating this process for all *n* parts and averaging the results provides robust results that represent the set from which the sample was drawn and an estimate of the accuracy that can be expected for unknown data from a similar sample.

We decided to use an elastic net as a machine learning model^73^. Elastic net combines the advantages of both Ridge regression^74^ and Lasso^75^ by penalizing not only the sum of squares error—as most linear models do—but also the squared sum of the coefficients (as Ridge regression does) and the absolute sum of the coefficients (as Lasso does). The squared penalization reduces the impact of unimportant features, whereas the absolute penalty leads to a model with fewer overall features. This means that our model was resistant to overfitting our data and should offer a good level of generalization. The variant of elastic net that we used offered built-in parameter optimization with cross-validation, so parameters for the weighing of the two penalty terms did not have to be set by hand. Whereas regularization helps prevent overfitting, cross-validation can indicate whether overfitting did in fact take place. If a model were overfit to a training set, the results (e.g., explained variance) when evaluating the model using the test set would suffer. The combined use of these two methodological tools affirmed our confidence in the robustness and generalizability of our results.

Another advantage of our machine learning approach was its ability to estimate feature weights. These feature weights indicated an individual feature’s contribution to the model and, in turn, allowed us to draw conclusions about a specific feature’s importance. This process helped us remove the “black box” character of the machine learning model. Feature weights are to be interpreted relative to each other and are similar to absolute values of regression coefficients. The feature weights that we provide are like unstandardized regression coefficients from a linear regression and can be interpreted in the same way. The machine learning model does not offer an indicator of statistical significance (i.e., *p*-values) for individual features/variables; however, any nonzero coefficients contribute to the fit of the model. As these coefficients are generated through cross-validation, they generalize within our population. We reran the analyses reported above using the Big Five facets as predictors.

#### Predicting CFT scores from gaze patterns (RQ2)

The analyses for RQ2 involved three parts: (a) preprocessing of the gaze data, (b) defining scanpath similarity, and (c) predicting CFT scores on the basis of the pairwise similarity of scanpaths.

##### Preprocessing of the gaze data

As a first step in investigating gaze patterns, we divided the stimulus (i.e., each item from the test) into areas of interest that corresponded to their semantic function, meaning that we focused on questions, targets, distractors, and others (including the clock and progress bar). Reducing the specific answer options a, b, c, etc. to semantic information helped us better understand participants’ gaze behavior independently of the specific item by revealing strategies that might not rely on the ordering of targets and distractors. Second, we normalized all scanpaths along their temporal axes. By doing so, we disregarded the duration of trials and focused purely on the strategic approach demonstrated by the participants. Without this normalization step, the length of a scanpath would indirectly encode how long participants took to arrive at a decision, trivializing the problem to a certain extent, as using efficient gaze patterns helps participants solve items more quickly and with a higher success rate. The normalized scanpaths were then binned into 50 equidistant temporal bins for the next steps in the analysis.

##### Defining scanpath similarity

We next defined scanpath similarity. Defining a meaningful similarity metric between scanpaths yielded a twofold benefit: On the one hand, it allowed us to test whether participants with similar gaze behavior had similar CFT scores. On the other hand, it allowed us to group similar participants and investigate what makes their behavior similar. We used a variation of the Needleman-Wunsch algorithm^76^ to conduct a pairwise comparison of participants’ scanpaths^77^. This algorithm is in essence a global string-alignment algorithm that applies an element-wise comparison metric to find the best matching substring for two given strings. If we define a scanpath as a sequence of eye positions on areas of interest, we arrive at a very similar structure that allows us to apply the Needleman-Wunsch algorithm to it. In our case, elements were gaze positions in areas of interest (e.g., questions, targets, distractors), and the algorithm looked for a scanpath that best represented the two scanpaths that were being compared. The length of this best-aligned subpath was indicative of the similarity of the two scanpaths. If it was very short, the two compared scanpaths were very dissimilar, whereas a long “common” path indicated that the two scanpaths were very similar. We decided to use a global alignment algorithm instead of a local approach, as our normalization step made the two identical, and global alignment is a specialized form that computes more quickly. Our variation of the Needleman-Wunsch algorithm used a simple scoring system, where gap cost and mismatch cost were set to −1, and matches got a score of 1. This resulted in the following scoring matrix, which can be iteratively computed:

1 $${M}_{{ij}}=\left\{\begin{array}{c}M\left(i-1,j-1\right)+\omega ({a}_{i},{b}_{j}),{\rm{\& }}{Match\; or\; Mismatch}\\ M\left(i-1,j\right)-1,{\rm{\& }}{Deletion}\\ M\left(i,j-1\right)-1,{Insertion}\\ \qquad\qquad\qquad\qquad0,{No\; similarity}\end{array}\right.$$

Let $s{c}_{i}$ and $s{c}_{j}$ be two scanpaths *i* and *j*, and let ${M}_{{ij}}$ be the scoring matrix output by our algorithm. Then the similarity score for *i* and *j* is calculated as follows:

2 $${sim}\left(i,j\right)= max \left({M}_{{ij}}\right)/50$$

##### Predicting CFT scores from the pairwise similarity of scanpaths

**Fig. 5:**
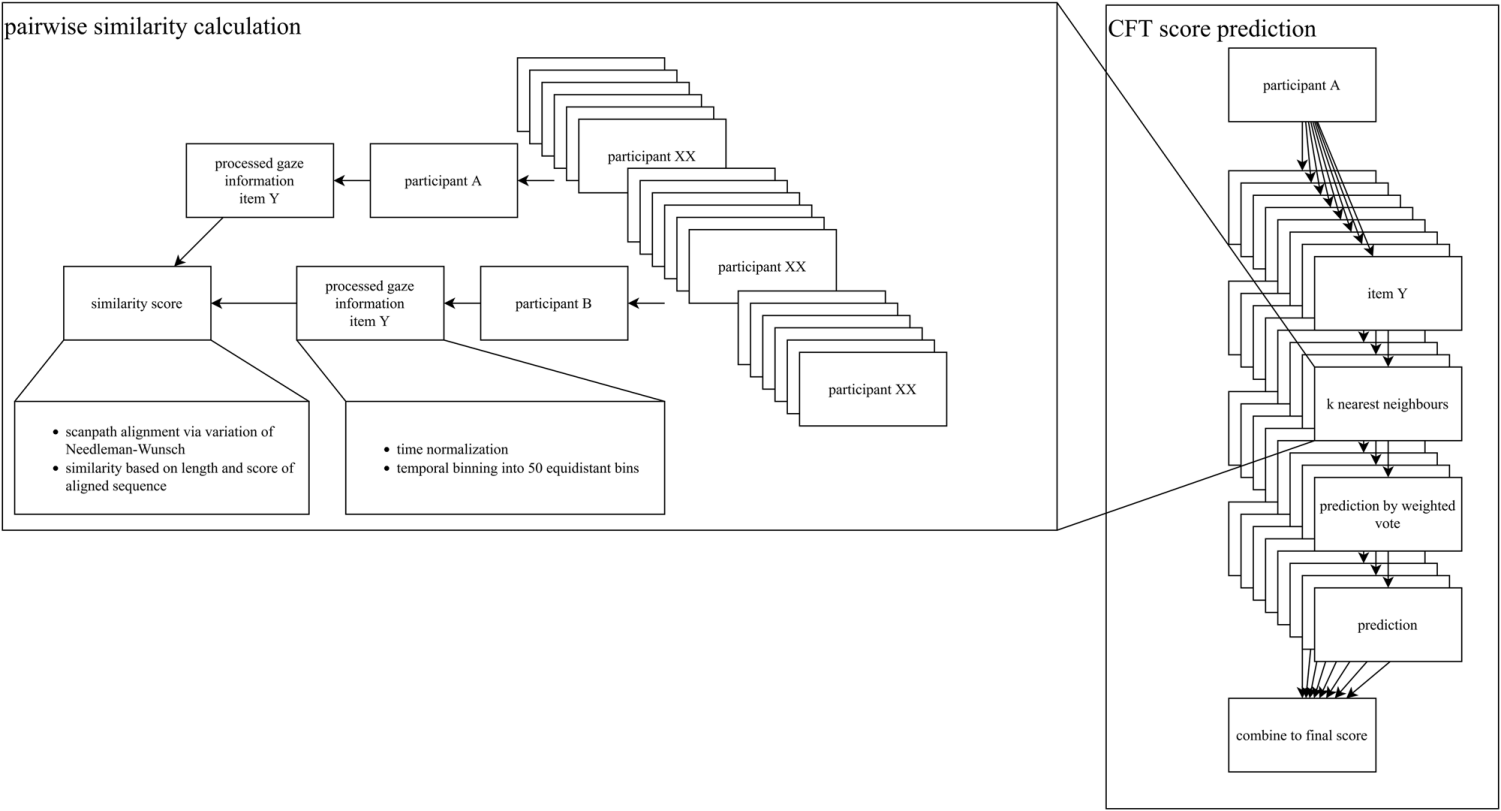
Predicting CFT scores from gaze patterns. Graphical representation of the prediction of CFT scores from the pairwise similarity of scanpaths.

We used the calculation of similarity between scanpaths to make predictions for CFT scores (see Fig. 1). To this end, we first predicted the probability that participant *p* solved item *i* correctly by looking at the *n* = 5 participants most similar in gaze behavior to participant *p* while solving item *i*. We weighted their success (0 or 1) for this item by their relative similarity to *p* and arrived at a predicted probability between 0 and 1. This process was iterated for all items and participants. Finally, we aggregated these predictions into a final score by adding them up on the participant level. A schematic description of our approach can be seen in Fig. 5. We decided on *n* = 5 in order to focus only on the most similar participants and not unnecessarily broaden the scope of our approach. Additionally, *n* = 5 yielded the most explained variance of all choices of *n*.

The following formula describes how the final predictions were calculated from the similarity matrix M:

3 $${pred}\left(p,i\right)=\frac{\sum {sim}\left({pat}{h}_{p,i},{pat}{h}_{q,i}\right){correct}\left(q,i\right)}{\sum _{q{\rm{\epsilon }}{P}_{p,k}}{sim}\left({pat}{h}_{p,i},{pat}{h}_{q,i}\right)}$$

where:

- $p\in P$ a participant in our study
- $q\in {P}_{p,k}$ one of the *k* participants most similar to *p*
- ${pat}{h}_{p,i}$ the scanpath of participant *p* while solving item *i*
- ${sim}\left({pat}{h}_{1},{pat}{h}_{2}\right)$ the similarity between scanpaths ${pat}{h}_{1}$ and ${pat}{h}_{2}$
- ${corect} ({q},{i})$ indication of whether participant *q* solved item *i*

As data from all but one participant were used to make a prediction, and we got a prediction for only one participant from this method, this approach lends itself particularly well to a leave-one-participant-out cross-validation. Leave-one-participant-out cross-validation refers to training a model on all but one participant and evaluating the model on the one participant who had been withheld. It also means that we strictly separated the data that generated the prediction from the data that we evaluated it on.

#### Predicting personality traits from the pairwise similarity of scanpaths (RQ3)

We used the same *k*-nearest-neighbor approach that relied on scanpath similarities as we did for the prediction of CFT scores from scanpaths (including the same leave-one-participant-out cross-validation). To arrive at predictions for personality traits, we averaged the predictions for each participant across the items on the test. This allowed us to investigate whether the use of strategies was connected to personality traits. Furthermore, we also investigated a model that used items only when the CFT predictions based on gaze were significantly above the level of chance (see the Predicting CFT Scores From the Pairwise Similarity of Scanpaths section). This allowed us to shed light on the question of whether personality traits are connected to gaze patterns that are indicative of intelligence test performance. The analyses that used personality facets were performed in the same way as the analyses that used the Big Five dimensions.

#### Extracting strategies (RQ4)

To get a better understanding of the presented similarity metric and the implicit strategies it encodes, we had initially planned to label representatives of common strategies for solving intelligence test items from the literature (constructive matching and response elimination) by hand and use them as a foundation for either the clustering or classification of those strategies. However, it proved difficult to find sufficiently clear examples for both strategies with the data for each item, as the strategies in our data were in many cases situated between the two extremes of constructive matching and response elimination, especially for challenging test items. Furthermore, declaring that specific gaze patterns reflected certain strategies, even if they did not fit exactly, would have introduced bias. Therefore, we adopted a different approach. We devised an iterative method to look into the most representative scanpaths for each question on the test. For each question *i*, each participant *p* was assigned a score on the basis of how many participants had participant *p* as one of their *n* = 11 most similar scanpaths. Subsequently, the participant *p* with the highest score was chosen as the representative of a strategy for solving this question, and all participants that had *p* as one of their most similar scanpaths were excluded in the next iteration. This process was repeated three times to choose the three scanpaths that provided the best possible representation of all participants’ behavior. The choice of *n* = 11 (as opposed to *n* = 5 in other parts of our method) was made on the basis of our observation that lower numbers tended to produce scanpaths that were very similar and, as such, did not provide much information. Therefore, *n* = 11 was identified as a good tradeoff between representative scanpaths that were not too broad (i.e., representing a wide variety of scanpaths) and not too narrow (i.e., only representing a small and very specific subset of scanpaths) because it produced diverse scanpaths within the first three iterations. The description in pseudocode is as follows:

Let ${M}_{t}$ be the similarity matrix for task $t$ with entries ${M}_{t}\left(p,q\right)$ corresponding to the similarity between participants $p$ and $q$.

$$k=1{while}\,k\le 3:$$

calculate the sum for participant $p$ as ${\sum }_{q}({M}_{t}(p.q) \, > \, 0) \,\forall q$

take ${p}_{\max }$ with the highest result

get the scanpath of ${p}_{\max }$ for task *t*

set ${M}_{t}({q,p}_{max })=0$, which removes ${p}_{max }$ from the pool and all pointers to it

$$k=k+1$$

Moreover, to better contextualize the findings resulting from this approach, a few explanations about the most representative scanpaths are needed. First, because scanpaths were time-normalized, length could be disregarded. Second, being a representative path just meant that it was similar to many other scanpaths and thus seemed to represent this group of scanpaths. Consequently, a high success rate for scanpaths that were similar did not necessarily mean that the representative path itself belonged to a participant who solved this specific item successfully. Furthermore, other scanpaths were just similar, but not identical, so individual behavior and strategy may vary even within this group.

#### Predicting CFT scores by combining gGaze information and personality traits (RQ5)

Finally, we refined the predictions for intelligence test scores described in the previous section (see the Predicting CFT Scores from the Pairwise Similarity of Scanpaths section) by adding participants’ personality traits. Including their traits provided information about the extent to which personality predicts intelligence test scores, above and beyond effects of gaze patterns. To this end, we relied on a machine learning regression model that used personality traits and gaze-based predictions of CFT scores to cast a refined prediction of CFT scores. To be flexible and accommodate the uncertain nature of the connection between gaze-based information, personality, and intelligence test scores, we decided to use the same elastic net method as we did to predict CFT scores on the basis of personality traits alone. With its dual regularization parameters, an elastic net should be well-suited to model this relationship. To ensure a fair weight for all features, and to avoid having features that were numerically larger dominating the prediction, features were normalized. Additionally, we used a loop with cross-validation to fit the regularization parameters for the model. This loop served the purpose of finding suitable parameters for the model but not overfitting them to the data, thereby ensuring robust results. Finally, results were reported with 10-fold cross-validation, which is a common approach for this kind of machine learning task.

The same approach was used when predicting intelligence test scores based on gaze and facet-level personality traits.

### Reporting summary

Further information on research design is available in the Nature Research Reporting Summary linked to this article.

### Supplementary information

Supplemental Material

Reporting summary

## Data Availability

We describe the sample and procedure and report all data exclusions in detail. We performed a secondary data analysis of data from the TüEyeQ project (see ref. ^27^; see also e.g., refs. ^66,77^). The eye-tracking data are publicly available at https://dataverse.harvard.edu/dataset.xhtml?persistentId=doi:10.7910/DVN/JGOCKI. The personality data has not yet been made publicly available but can be obtained from the TüEyeQ project team.

## Code availability

All analysis codes are available on the Open Science Framework (OSF: https://osf.io/34bm8/). Data were analyzed with Python and the scikit-learn package.
