# Supplementary material for: Taking another look at intelligence and personality using an eye-tracking approach
Source: NPJ Sci Learn. 2024 Jul 1;9:41. doi: 10.1038/s41539-024-00252-8 (PMC11217503; doi:10.1038/s41539-024-00252-8)
Supplement: Supplementary file 1 — Supplemental Material [file 41539_2024_252_MOESM1_ESM.pdf]

Online Supplement

Supplementary Table 1

Descriptive Statistics and Bivariate Correlation Coefficients for Big Five Facets and Intelligence Test Scores

[Note that this is uploaded as excel-file to OSF due to the large number of correlations: https://osf.io/34bm8/*]*

Supplementary Table 2

Results for RQ1 (Big Five Personality Traits Predicting Intelligence Test Performance) Using A Standard (Multiple) Regression

Variable	*B*	95% CI	*t*	*p*
Constant	0.7294	[0.547, 0.912]	7.889	< 0.001***
Neuroticism	-0.0184	[-0.047, 0.010]	-1.282	0.202
Extraversion	-0.0159	[-0.051, 0.019]	-0.897	0.371
Openness	0.0497	[0.005, 0.094]	2.201	0.029*
Agreeableness	0.0417	[0.004, 0.079]	2.188	0.030*
Conscientiousness	-0.0181	[-0.053, 0.017]	-1.030	0.305

Note. $N = 182$. CI = Confidence interval. $R^2_{adj} = .054$, $p = .0106$. $F(5, 176) = 3.081$.
*$p < .05$. **$p < .01$. ***$p < .001$. Two-tailed tests were conducted.

Supplementary Table 3

Results for RQ1, with Big Five Personality Traits Predicting Intelligence Test Performance Using Machine Learning and Relying on the Entire Sample (*N* = 315)

Variable	Coefficient
Neuroticism	0
Extraversion	0
Openness	0.0686
Agreeableness	0.0338
Conscientiousness	0

Note. Total explained variance = 0.0780.

Supplementary Table 4

Additional Demographic Information of the Sample Used in the Present Study (*N* = 182)

Variable	Number (Percentage)
Percentage Native German speakers	174 (95.58%)
Primary study subject	
Arts and humanities	43 (23.63%)
Business, administration and law	23 (12.64%)
Education	28 (15.38%)
Engineering, manufacturing and construction	0 (0.00%)
Generic programs and qualifications	0 (0.00%)
Health and welfare	13 (7.14%)
Information and communication technologies	5 (2.75%)
Natural sciences, mathematics and statistics	21 (11.54%)
Services	2 (1.10%)
Social sciences, journalism and information	46 (25.27%)
N/A	1 (0.55%)
Highest scholarly or professional education of the participant’s mother	
No completed education	4 (2.20%)
Completed apprenticeship or graduated from a vocational school	0 (0.00%)
Graduation from a vocational school or commercial school	17 (9.34%)
Graduation from a technical school or health care school	66 (36.26%)
Degree from a university of applied sciences or degree from a cooperative education institution	8 (4.40%)
University degree (master's degree, diploma, state examination, doctorate)	81 (44.51%)
Other degree	0 (0.00%)
N/A	6 (3.30%)
Highest scholarly or professional education of the participant’s father	
No completed education	0 (0.00%)
Completed apprenticeship or graduated from a vocational school	0 (0.00%)
Graduation from a vocational school or commercial school	26 (14.29%)
Graduation from a technical school or health care school	37 (20.33%)
Degree from a university of applied sciences or degree from a cooperative education institution	18 (9.89%)

University degree (master's degree, diploma, state examination, doctorate)	93 (51.10%)
Other degree	0 (0.00%)
N/A	8 (4.40%)
Number of books in the participant’s household	
0-10	5 (2.75%)
10-25	7 (3.85%)
26-100	37 (20.33%)
101-200	44 (24.18%)
201-500	54 (29.67%)
> 500	34 (18.58%)
N/A	1 (0.55%)

Note. N/A = Not answered or not available.
