# Supplementary material for: Taking another look at intelligence and personality using an eye-tracking approach
Source: NPJ Sci Learn. 2024 Jul 1;9:41. doi: 10.1038/s41539-024-00252-8 (PMC11217503; doi:10.1038/s41539-024-00252-8)
Supplement: Supplementary file 2 — Reporting summary [file 41539_2024_252_MOESM2_ESM.pdf]

nature portfolio

Corresponding author(s): *Double-anonymous peer review submissions: write DAPR and your manuscript number here instead of author names.*

Last updated by author(s): *YYYY-MM-DD*

Reporting Summary

Nature Portfolio wishes to improve the reproducibility of the work that we publish. This form provides structure for consistency and transparency in reporting. For further information on Nature Portfolio policies, see our Editorial Policies and the Editorial Policy Checklist.

Statistics

For all statistical analyses, confirm that the following items are present in the figure legend, table legend, main text, or Methods section.

n/a	Confirmed	
☐	☒	The exact sample size (*n*) for each experimental group/condition, given as a discrete number and unit of measurement
☐	☒	A statement on whether measurements were taken from distinct samples or whether the same sample was measured repeatedly
☐	☒	The statistical test(s) used AND whether they are one- or two-sided<br>*Only common tests should be described solely by name; describe more complex techniques in the Methods section.*
☒	☐	A description of all covariates tested
☐	☒	A description of any assumptions or corrections, such as tests of normality and adjustment for multiple comparisons
☐	☒	A full description of the statistical parameters including central tendency (e.g. means) or other basic estimates (e.g. regression coefficient) AND variation (e.g. standard deviation) or associated estimates of uncertainty (e.g. confidence intervals)
☐	☒	For null hypothesis testing, the test statistic (e.g. *F*, *t*, *r*) with confidence intervals, effect sizes, degrees of freedom and *P* value noted<br>*Give P values as exact values whenever suitable.*
☒	☐	For Bayesian analysis, information on the choice of priors and Markov chain Monte Carlo settings
☒	☐	For hierarchical and complex designs, identification of the appropriate level for tests and full reporting of outcomes
☐	☒	Estimates of effect sizes (e.g. Cohen's *d*, Pearson's *r*), indicating how they were calculated

Our web collection on statistics for biologists contains articles on many of the points above.

Software and code

Policy information about availability of computer code

Data collection	We used the publicly available TüEyeQ data set (Kasneci et al., 2021), which is part of a larger scale lab study on self-regulation (see Kasneci et al., 2021, for details; see also Kasneci et al., 2022). The TüEyeQ data set contains eye movement data from 315 university students while they solved the first part of the CFT 20-R, an intelligence test employing figural tasks (Weiß et al., 2006) and sociodemographic data. We further enriched this data set with information on the Big Five (available upon request from the TüEyeQ project team). All data were collected throughout 2018, beginning in February and finishing in December, in a sample of adults with a university entrance qualification, no preexisting neurological or psychiatric conditions, and no visual impairment above 3 diopters. All tests and questionnaires were administered on a 17-inch laptop with a resolution of 1920 x 1080. The eye trackers were SMIred manufactured by SMI and run at 250Hz. Calibration was performed with a 9-point calibration before each test and questionnaire, and lighting conditions were kept constant throughout each recording session and across sessions. Participants could choose whether they wanted to use a mouse or the built-in touchpad for their interactions with the laptop (see also Kasneci et al., 2021).
Data analysis	Data were analyzed with Python and the scikit-learn package. All analysis codes are available on the Open Science Framework (OSF; anonymized link for peer review: https://osf.io/34bm8/?view_only=9e721aa468ab4d739488ca1e976f78cf).

For manuscripts utilizing custom algorithms or software that are central to the research but not yet described in published literature, software must be made available to editors and reviewers. We strongly encourage code deposition in a community repository (e.g. GitHub). See the Nature Portfolio guidelines for submitting code & software for further information.

Data

Policy information about availability of data

All manuscripts must include a data availability statement. This statement should provide the following information, where applicable:

- Accession codes, unique identifiers, or web links for publicly available datasets
- A description of any restrictions on data availability
- For clinical datasets or third party data, please ensure that the statement adheres to our policy

Data were analyzed with Python and the scikit-learn package. We performed a secondary data analysis of data from the TüEyeQ project (see Kasneci et al., 2021; see also e.g., Appel et al., 2022; Kasneci et al., 2022). The eye-tracking data are publicly available at https://dataverse.harvard.edu/dataset.xhtml?persistentId=doi:10.7910/DVN/JGOCKI. The personality data can be obtained from the TüEyeQ project team for research purposes.

Research involving human participants, their data, or biological material

Policy information about studies with human participants or human data. See also policy information about sex, gender (identity/presentation), and sexual orientation and race, ethnicity and racism.

Reporting on sex and gender	In our study, gender was assessed using self-reports. No information on sex was collected. We did not perform gender-based analyses as we had no research question involving gender.
Reporting on race, ethnicity, or other socially relevant groupings	Information on whether participants were native German speakers was collected using self-reports (almost 96% of our sample were German native speakers). No other information on ethnicity, race, or cultural background was collected. Self-reported information on the levels of education of the participants' parents was also collected. We did perform analyses including native language and parental education as we did not have research questions involving these variables.
Population characteristics	See above
Recruitment	Participants (university students) were recruited to participate in the research project at Tübingen. We cannot rule out that individuals who were more interested in research etc. were more likely to participate.
Ethics oversight	The Ethics Committee at the Psychological Institute at the University of Tübingen confirmed that the procedures fulfilled all ethical standards of research with human subjects.

Note that full information on the approval of the study protocol must also be provided in the manuscript.

Field-specific reporting

Please select the one below that is the best fit for your research. If you are not sure, read the appropriate sections before making your selection.

☐ Life sciences ☒ Behavioural & social sciences ☐ Ecological, evolutionary & environmental sciences

For a reference copy of the document with all sections, see nature.com/documents/nr-reporting-summary-flat.pdf

Behavioural & social sciences study design

All studies must disclose on these points even when the disclosure is negative.

Study description	Quantitative study including questionnaires, an intelligence test and behavioral data (while participants completed the intelligence test)
Research sample	We used the publicly available TüEyeQ data set (Kasneci et al., 2021), which is part of a larger scale lab study on self-regulation (see Kasneci et al., 2021, for details; see also Kasneci et al., 2022). The TüEyeQ data set contains eye movement data from 315 university students while they solved the first part of the CFT 20-R, an intelligence test employing figural tasks (Weiß et al., 2006) and sociodemographic data. We further enriched this data set with information on the Big Five (available upon request from the TüEyeQ project team). The final sample consisted of 182 participants (Mage = 23.32, SDage = 2.89; 71.82% women) for our analysis (see also Kasneci et al., 2021). This was not a representative sample.
Sampling strategy	We conducted a secondary data analysis of an already existing data set (see above).
Data collection	The data was collected in a digital classroom. The classroom was equipped with 30 remote eye trackers attached to laptops with 17inch HD display screens running at full brightness. This setup allows for data collection of up to 30 participants simultaneously, which minimizes the overall time needed for collection. Verbal instructions were given to the entire group (a brief overview of the protocol and an explanation of eye tracking), then individual calibrations were performed with a supervised quality check. The participants interacted with the computer took place via mouse or touch pad, depending on participants' preference. The collection environment controlled the room illumination level, which ensured no effects from sunlight or other outdoor light. The standard maintained illuminance for the experimental sessions ranged between 10 to 50 lux, and was measured with a Lux sensor (i.e., Gossen Mavo-Max illuminance sensor, MC Technologies, Hannover, Germany). All instructions were in German using the SoSci Survey online

platform (Kasneci et al., 2021).

Timing	All data were collected throughout 2018, beginning in February and finishing in December.
Data exclusions	After excluding 86 participants' eye-tracking data for various reasons (58 because of a poor tracking rate [below 80%], 11 due to errors during the presentation of our stimulus, and 17 due to incomplete data), data from 229 participants remained. As the eye movements of several participants did not meet the accuracy standards required for our analyses, 43 more participants were excluded after manual inspection (e.g., if it was obvious that a participant's gaze did not match the stimulus at all, indicating movement after calibration that tends to lead to large offsets in x and y coordinates). These 43 participants showed substantial offsets in their fixation location, likely caused by head movements after calibration, and rendering a reliable matching of fixations to areas of interest impossible. In addition, another four participants were not considered because their data from different tasks could not be matched reliably, or their eye-tracking data could not be paired with participants' intelligence test performance data. The final sample consisted of 182 participants (Mage = 23.32, SDage = 2.89; 71.82% women) for our analysis.
Non-participation	This is a secondary data analysis of an existing data set; no drop-out was reported (but see above for information on data exclusions).
Randomization	N/A

Reporting for specific materials, systems and methods

We require information from authors about some types of materials, experimental systems and methods used in many studies. Here, indicate whether each material, system or method listed is relevant to your study. If you are not sure if a list item applies to your research, read the appropriate section before selecting a response.

Materials & experimental systems

n/a	Involved in the study
☒	☐ Antibodies
☒	☐ Eukaryotic cell lines
☒	☐ Palaeontology and archaeology
☒	☐ Animals and other organisms
☒	☐ Clinical data
☒	☐ Dual use research of concern
☒	☐ Plants

Methods

n/a	Involved in the study
☒	☐ ChIP-seq
☒	☐ Flow cytometry
☒	☐ MRI-based neuroimaging
